# Comparison of the risk factors effects between two populations: two alternative approaches illustrated by the analysis of first and second kidney transplant recipients

**DOI:** 10.1186/1471-2288-13-102

**Published:** 2013-08-06

**Authors:** Katy Trébern-Launay, Magali Giral, Jacques Dantal, Yohann Foucher

**Affiliations:** 1 Department of Biostatistics EA 4275, Clinical Research and Subjective Measures in Health Sciences, University of Nantes, 1 rue Gaston Veil, Nantes 44035, France; 2 Transplantation, Urology and Nephrology Institute (ITUN), Nantes Hospital and University, Inserm U1064, 30 bd Jean Monnet, Nantes 44093, France

## Abstract

**Background:**

Whereas the prognosis of second kidney transplant recipients (STR) compared to the first ones has been frequently analyzed, no study has addressed the issue of comparing the risk factor effects on graft failure between both groups.

**Methods:**

Here, we propose two alternative strategies to study the heterogeneity of risk factors between two groups of patients: *(i)* a multiplicative-regression model for relative survival (MRS) and *(ii)* a stratified Cox model (SCM) specifying the graft rank as strata and assuming subvectors of the explicatives variables. These developments were motivated by the analysis of factors associated with time to graft failure (return-to-dialysis or patient death) in second kidney transplant recipients (STR) compared to the first ones. Estimation of the parameters was based on partial likelihood maximization. Monte-Carlo simulations associated with bootstrap re-sampling was performed to calculate the standard deviations for the MRS.

**Results:**

We demonstrate, for the first time in renal transplantation, that: *(i)* male donor gender is a specific risk factor for STR, *(ii)* the adverse effect of recipient age is enhanced for STR and *(iii)* the graft failure risk related to donor age is attenuated for STR.

**Conclusion:**

While the traditional Cox model did not provide original results based on the renal transplantation literature, the proposed relative and stratified models revealed new findings that are useful for clinicians. These methodologies may be of interest in other medical fields when the principal objective is the comparison of risk factors between two populations.

## Background

In patients facing a first allograft loss, repeat kidney transplantation provides a better chance for both long-term survival and quality of life than a return to dialysis [1,2]. The prognosis of second kidney transplant recipients (STR) compared to first kidney transplant recipients (FTR) has been frequently studied. The older literature tends to conclude that STR have a worse prognosis than FTR [3,4]. However, recent analyses with adjustments for confounding factors have challenged this generally accepted idea [5,6], with the exception of one study [7]. By modelling the time-dependent hazard between FTR and STR, we recently demonstrated that STR have a higher risk of graft failure than FTR, but this excess risk appears several years after transplantation [8]. According to this literature, one can accept that the excess risk for STR compared to FTR is negligible considering the improvements in life expectancy and quality of life compared to dialysis therapy. Nevertheless, as the demand for kidney transplants largely exceeds the supply [9], it is necessary to evaluate the differences in risk factors between STR and FTR so as to improve graft allocation.

For this purpose, traditional survival models can be used by merging STR and FTR. Nevertheless, one can notice two important limitations of these traditional approaches. Firstly, the comparisons of risk factors between both groups would imply testing all the interactions with the graft rank. Secondly, STR-specific explicative variables (survival time of the first transplant, transplantectomy or time in dialysis before re-transplantation) cannot be included despite the knowledge that their use would improve risk evaluation [5,6,10,11].

To overcome these limits, in this paper we propose two alternative strategies. The first one is an adaptation of a multiplicative-regression model for relative survival (MRS). This type of relative approach is often used to study the net survival of patients with cancer, i.e. the survival of patients if the only cause of death is related to the disease [12-17]. The principle of such additive-regression models is to introduce expected mortality rates by using life tables adjusted for gender, age and calendar year. Still using life tables, Andersen et al. [18] proposed a multiplicative-regression model. To our knowledge, the development of such methodology to endpoints other than mortality and with a reference group without a life time, has never been explored.

Moreover, we propose a second method by adapting a stratified Cox model (SCM) specifying the graft rank as strata and assuming a vector of explicative variables decomposed into subvectors of variables that enter either in the reference hazard only, or in the relative hazard only, or in both groups but with common or separate effects.

## Methods

### Study population

Second transplant recipients (STR) constituted the relative group of interest. Recipients older than 18 years at the date of transplantation between 1996 and 2010 were selected from the French DIVAT (Données Informatisées et VAlidées en Transplantation - http://www.divat.fr/en) multicentric prospective cohort [19]. Codes were used to assure donor and recipient anonymity and blind assay. The ’Comité National Informatique et Liberté’ approved the study (N° CNIL 891735) and written informed consent was obtained from the participants. Only recipients with a maintenance therapy with calcineurin inhibitors, mammalian target of rapamycin inhibitors or belatacept, in addition to mycophenolic acid and steroids, were included. Simultaneous transplantations were excluded. Recipients with at least one missing data for all the variables taken into account in the expected hazard were excluded. The same criteria were applied to the reference group composed of first transplant recipients (FTR). The principal outcome was the time between transplantation and graft failure, which was the first event between return to dialysis and patient death with a functioning graft.

### The multiplicative-regression model for relative survival (MRS)

Let the individuals be indexed by *j* (*j* = 1,...,*n*_*e*_) in the reference group and by *i* (*i* = 1,...,*n*_*r*_) in the relative group. *n*_*e*_ and *n*_*r*_ represent the sample sizes of the respective groups. We note *h*^*o*^(*t*_*i*_|*z*_*i*_) the observed instantaneous hazard function at time *t*_*i*_ for the *i*th individual, where *z*_*i*_ is the vector of explicative variables. The observed hazard of the *i*th subject of the relative group can be decomposed in the multiplication of two hazards [18,20]

(1) $$h^{o}(t_{i}|z_{i})=h^{e}(t_{i}|z_{i}^{e})\;h^{r}(t_{i}|z_{i}^{r})$$

where $h^{e}(t_{i}|z_{i}^{e})$ is the expected hazard for an individual in the reference group with similar characteristics to the *i*th individual. $z_{i}^{e}$ is a subset of *z*_*i*_ and represents these common characteristics. $h^{r}(t_{i}|z_{i}^{r})$ is the relative hazard with $z_{i}^{r}$ being a subset of *z*_*i*_.

Parameters of the expected hazard can be estimated assuming a semi-parametric and proportional hazards (PH) model [21]. For the *j*th individual (*j* = 1,...,*n*_*e*_)

(2) $$h^{e}(t_{j}|z_{j}^{e})=h_{0}^{e}(t_{j})\text{exp}(\beta^{e}z_{j}^{e})$$

where $h_{0}^{e}(t_{j})$ is an unknown expected baseline hazard function and *β*^*e*^ is the vector of regression coefficients associated with $z_{j}^{e}$. The estimations ${\hat{\beta }}^{e}$ are obtained by maximizing the partial log-likelihood among the *n*_*e*_ patients of the reference group

(3) $$\;\text{log}{Pℒ}_{e}(\beta^{e})=\overset{n_{e}}{\underset{j=1}{\sum }}\delta_{j}\left\{\beta^{e}z_{j}^{e}-\text{log}\left(\underset{k:t_{k}\geq t_{j}}{\sum }\text{exp}(\beta^{e}\overset{e}{\underset{k}{z}})\right)\right\}$$

where *δ*_*j*_ equals 1 if the failure was observed for the *j*th subject and 0 otherwise. The estimation of the variance-covariance matrix, $\hat{V}({\hat{\beta }}^{e})$, is obtained via the corresponding information matrix.

Parameters of the relative hazard can also be estimated using a semi-parametric PH model. For the *i*th individual (*i* = 1,...,*n*_*r*_), the instantaneous hazard is defined as

(4) $$h^{r}(t_{i}|z_{i})=h_{0}^{r}(t_{i})\text{exp}(\beta^{r}z_{i}^{r})$$

where $h_{0}^{r}(t_{i})$ is an unknown relative baseline hazard function and *β*^*r*^ is the vector of regression coefficients associated with $z_{i}^{r}$. By adapting the partial likelihood function (3) and assuming the previous estimations of expected parameters as constants, the regression coefficients *β*^*r*^ are estimated by maximizing

(5) $$\begin{array}{ll} \;\text{log}{Pℒ}_{r}(\beta^{r}) & =\overset{n_{r}}{\underset{i=1}{\sum }}\delta_{i}\left\{{\hat{\beta }}^{e}z_{i}^{e}+\beta^{r}z_{i}^{r}\right)\\ & \;\;\;\;\;\left(-\text{log}\left(\underset{k:t_{k}\geq t_{i}}{\sum }\text{exp}({\hat{\beta }}^{e}\overset{e}{\underset{k}{z}})\right)\right\} \end{array}$$

Of note, for explicative variables not taken into account in the expected hazard in the model (1), exp(*β*^*r*^) represents the observed hazard ratios (HR) in the reference group, just as exp(*β*^*r*^) estimated from the PH model (4) among the relative group. In contrast, for explicative variables taken into account in the expected hazard model in the model (1), exp(*β*^*r*^) represents the weighting factors between the expected HR, i.e. $\text{exp}({\hat{\beta }}^{e})$, and the observed HR in the relative group, i.e. $\text{exp}({\hat{\beta }}^{e})\times \text{exp}(\beta^{r})$. In other words, for explicative variables involved in both models (2) and (4), *β*^*r*^ = 0 means that the variable has the same effect in both groups. If *β*^*r*^ > 0, the hazard ratio increases in the relative group compared to the reference group. If *β*^*r*^ < 0, the hazard ratio decreases.

Nevertheless, in contrast to the traditional relative survival models based on life tables, the expected hazards cannot be reasonably assumed as constants since the corresponding parameters were estimated from the reference sample. To take into account the variability associated with the expected model (2) in the estimation of the relative model (4), we used Monte-Carlo simulations associated with bootstrap resampling [22]. At each of the *B* iterations (b=1,…,B), this procedure can be divided into the following steps 

• Generation of a vector of parameters ${\hat{\beta }}_{b}^{e*}$ using the multivariate normal distribution $N\left({\hat{\beta }}^{e},\hat{V}({\hat{\beta }}^{e})\right)$ obtained from the maximisation of the partial likelihood (3). This first step takes into account the variance of the expected hazard.

• Generation of a bootstrap sample from the relative sample comprising *n*_*r*_ subjects. This second step takes into account the variance due to sample-to-sample fluctuation.

• From this bootstrap sample, the model (4) is estimated by maximizing (5) in which the simulated parameters ${\hat{\beta }}_{b}^{e*}$ are used instead of ${\hat{\beta }}^{e}$. ${\hat{\beta }}_{b}^{r}$ is the resulting estimation of the relative regression coefficients.

Means, standard deviations and 95% confidence intervals can be calculated from the *B* estimations of ${\hat{\beta }}_{b}^{e*}$.

### The stratified Cox model (SCM)

For the *i*th individual of the overall sample (*i* = 1,...,*n*), i.e. the reference and the relative group together (*n* = *n*_*e*_ + *n*_*r*_), let *z*_*i*_ be the vector of covariates that are applied to the model for either first or second transplant recipients. Let *k* be the indicator of the strata with *k* = *e* for the reference group and *k* = *r* for the relative group. The stratified Cox model is given by

(6) $$h_{k}(t_{i}|z_{i})=h_{k,0}(t_{i})\;\text{exp}(\beta z_{i});\;\;\;\text{for}\;k=e,\mathrm{r.}$$

with *h*_*k*,0_(*t*_*i*_) the baseline hazard function in the strata *k*. The vector *z*_*i*_ can be decomposed into four different subvectors: *(i)* the vector $z_{i}^{e}$ of explicative variables that enter in the reference hazard only (their values equal 0 if *k* = *r*); *(ii)* the vector $z_{i}^{r}$ of explicative variables that enter in the relative hazard only (their values equal 0 if *k* = *e*); *(iii)* the vector $z_{i}^{c}$ of explicative variables associated to both groups; and *(iv)* the vector $z_{i}^{s}$ a subvector of explicative variables included in $z_{i}^{c}$ but with separate effects. The model (6) can be developed as follows

(7) $$\begin{array}{ll} h_{k}(t_{i}|z_{i}) & =h_{k,0}(t_{i})\;\text{exp}(\beta^{e}z_{i}^{e}+\beta^{c}z_{i}^{c}+\beta^{s}z_{i}^{s}\delta_{\text{ir}}+\beta^{r}z_{i}^{r});\;\;\;\\ & \;\;\;\text{for}\;k=e,\mathrm{r.} \end{array}$$

with *β* = (*β*^*e*^,*β*^*c*^,*β*^*s*^,*β*^*r*^) the vector of regression coefficients and *δ*_*ir*_ equals 1 if the subject *i* belongs to the strata *k* = *r* and 0 otherwise. Then, for the reference group, we obtain

(8) $$h_{e}(t_{i}|z_{i})=h_{e,0}(t_{i})\;\text{exp}(\beta^{e}z_{i}^{e}+\beta^{c}z_{i}^{c})$$

and for the relative group, we obtain

(9) $$h_{r}(t_{i}|z_{i})=h_{r,0}(t_{i})\;\text{exp}(\beta^{c}z_{i}^{c}+\beta^{s}z_{i}^{s}+\beta^{r}z_{i}^{r})$$

Therefore, *exp*(*β*^*e*^) represents the HR associated with specific variables for the reference group. And *exp*(*β*^*r*^) represents the HR associated with specific variables for the relative group. For variables only included in *z*^*c*^ (and not in *z*^*s*^), *exp*(*β*^*c*^) represents the common HR in both groups associated with *z*^*c*^. For variables included in both *z*^*c*^ and *z*^*s*^, the HR associated with *z*^*c*^ equals *exp*(*β*^*c*^) in the reference group and *exp*(*β*^*c*^+*β*^*s*^) in the relative group. Thus, *exp*(*β*^*s*^) represents the weighting factors between the expected HR, i.e. *exp*(*β*^*c*^), and the observed HR in the relative group, i.e. *exp*(*β*^*c*^) × *exp*(*β*^*s*^).

### Evaluation of the proportional hazards assumption

In models (2), (4) and (7), hazard proportionality was checked for each explicative variable by plotting log-minus-log survival curves obtained by the Kaplan and Meier estimator [23] and by testing the scaled Schoenfeld residuals [24] separately in the reference and relative samples. If the observed hazard ratios are constant regardless of time in both groups, the ratio between both observed hazard ratios, i.e. the weighting factor *exp*(*β*^*r*^), will also be constant.

### Software

All statistical analyses were performed using R version 2.15.1 [25]. The proposed multiplicative-regression model for relative survival was implemented in an R package MRsurv available at http://www.divat.fr/en/softwares/mrsurv. The adapted stratified Cox model was implemented by using the R package survival (function coxph, option strata).

## Results

### Description of the cohort

641 STR potentially made up the relative group of interest, but 75 STR (11.7%) with missing data for explicative variables of the expected hazard were excluded. Finally, 566 STR were included in the group of interest. The mean follow-up was 3.1 years with a maximum of 13.1 years. During the observation period, 72 returns to dialysis and 34 deaths were observed. We identified 2462 FTR who met the inclusion criteria. We excluded 256 FTR (10.3%) with one missing data for at least one of the variables taken into account in the expected hazard model. Finally, 2206 FTR made up the reference group. The mean follow-up was 3.4 years with a maximum of 13.7 years. During the observation period, 191 returns to dialysis and 109 deaths were observed.

The demographic and baseline characteristics at the time of transplantation are presented in Table 1. Regardless of the group, the majority of patients received a transplant from a deceased donor and the recipient gender was comparable between groups. However, STR were younger and their transplants were provided by younger donors. Recurrent nephropathy, past history of cardiac disease, hepatitis and malignancy were more frequent among STR, but STR had less diabetes and were less likely to be obese at the time of transplantation. Compared to FTR, STR received better HLA-matched transplants, but their cold ischemia time was longer and they were more immunized against HLA class I and class II antigens (historical Panel Reactive Antibodies) than FTR. They were also more frequently exposed to induction therapy with a lymphocyte-depleting agent.

**Table 1:** **Demographic characteristics at the date of transplantation for *(i) * whole cohort and *(ii) * FTR and STR separately; the last three rows of the table concern covariates specific for STR**

	**All (N = 2772)**		**FTR (N = 2206)**		**STR (N = 566)**		
**Demographic characteristics**	**Number (%)**		**Number (%)**		**Number (%)**		**p-value**
Transplantation period < 2005	594	(21.4)	457	(20.7)	137	(24.2)	0.0806
Recipient ≥ 55 years of age	1175	(42.4)	994	(45.1)	181	(32.0)	<0.0001
Male recipient	1705	(61.5)	1362	(61.7)	343	(60.6)	0.6536
Recurrent causal nephropathy	906	(32.7)	666	(30.2)	240	(42.4)	<0.0001
History of diabetes	306	(11.0)	269	(12.2)	37	(6.5)	0.0002
History of hypertension	2263	(81.6)	1804	(81.8)	459	(81.1)	0.7545
History of vascular disease	352	(12.7)	272	(12.3)	80	(14.1)	0.2804
History of cardiac disease	903	(32.6)	686	(31.1)	217	(38.3)	0.0012
History of dyslipemia	799	(28.8)	661	(30.0)	138	(24.4)	0.0104
History of malignancy	228	(8.2)	147	(6.7)	81	(14.3)	<0.0001
History of hepatitis B or C	168	(6.1)	96	(4.4)	72	(12.7)	<0.0001
Recipient BMI ≥ 30 kg.m ^-2^	263	(9.5)	235	(10.7)	28	(4.9)	<0.0001
Positive anti-class I PRA	706	(25.5)	355	(16.1)	351	(62.0)	<0.0001
Positive anti-class II PRA	733	(26.4)	319	(14.5)	414	(73.1)	<0.0001
Donor ≥ 55 years of age	1172	(42.3)	973	(44.1)	199	(35.2)	0.0002
Deceased donor	2470	(89.1)	1940	(87.9)	530	(93.6)	0.0002
Donor serum creatinine ≥ 133 *μ*mol/l	342	(12.5)	279	(12.8)	63	(11.4)	0.3807
Positive donor EBV serology	2613	(94.3)	2087	(94.6)	526	(92.9)	0.1540
HLA-A-B-DR incompatibilities > 4	365	(13.2)	326	(14.8)	39	(6.9)	<0.0001
Cold ischemia time ≥ 24h	754	(27.2)	552	(25.0)	202	(35.7)	<0.0001
Lymphocyte-depleting induction	1223	(44.1)	793	(35.9)	430	(76.0)	<0.0001
First graft survival < 1 year	-	-	-	-	131	(24.1)	-
Waiting time before regraft ≥ 3 years	-	-	-	-	272	(49.8)	-
Transplantectomy of the first graft	-	-	-	-	220	(38.9)	-

p-values were obtained by using the Chi-square statistic.

Among FTR meeting the inclusion criteria, some patients were also part of the STR group as they had received two transplants during the observation period. These 37 patients, who were included in both cohorts, represented 2% and 7% of the FTR and STR groups respectively. Given the large number of explicative variables, it seemed reasonable to assume conditional independence of the two transplantations of a given patient. In order to validate this assumption, we performed a frailty Cox model [26] based on the 37 individuals who were included in both groups. The frailty term was assumed to be Gamma distributed. The variance of the random variable was estimated at 5.10 ^-9^ (p = 0.9948). Therefore, no intra-individual dependency was demonstrated. In order to validate the robustness of the results, we also performed both models after exclusion of the 37 STR also included in FTR. These results are presented in Additional files 1 and 2.

### Analysis of risk factors in the FTR sample

As previously illustrated in Table 1, it is well-established that FTR and STR are not intrinsically comparable. Thus, for the analysis of risk factors in the FTR population, adjustments were made *(i)* for all of the possible pre- or per-transplant immunological and non-immunological confounding factors according to experts and *(ii)* for all the baseline parameters differentially distributed between FTR and STR. All together, the expected hazard of graft failure was estimated according to recipient age and gender, causal nephropathy, comorbidities (including history of diabetes, hypertension, cardiac or vascular disease, dyslipemia, hepatitis B or C, and malignancy), obesity, pre-transplant immunization (panel reactive antibody, PRA) against class I and class II antigens), donor age, deceased or living donor status, Epstein-Barr Virus (EBV) serology, period of transplantation, level of HLA-A-B-DR mismatches, induction therapy and cold ischemia time. This modelling is explained in detail in the paper by Trébern-Launay et al. [8]. The final multivariate model in the reference group of FTR is presented in the first three columns of Table 2.

**Table 2:** **Multivariate Cox model for FTR and results of the MRS in the STR group : *(i) * the first three columns provides the results of the multivariate Cox model analysis of graft failure risk factors for FTR (N = 2206); *(ii) * the next three columns provide the results of the relative survival model based on 540 STR (26 recipients presenting missing data for the waiting time before re-transplantation were excluded)**

	**Cox model in**			**MRS in**		
	**the FTR group**			**the STR group**		
	**HR**	**95% CI**	**p-value**	**HR**	**95% CI**	**p-value**
**Variables entering in the model for FTR only**						
Causal nephropathy (recurrent / non recurrent)	1.24	0.96-1.59	0.0987	-	-	-
History of diabetes (positive / negative)	1.34	0.96-1.85	0.0819	-	-	-
History of hypertension (positive / negative)	0.77	0.57-1.05	0.0986	-	-	-
History of cardiac disease (positive / negative)	1.41	1.11-1.79	0.0051	-	-	-
History of vascular disease (positive / negative)	1.10	0.81-1.51	0.5351	-	-	-
History of dyslipemia (positive / negative)	1.12	0.87-1.45	0.3828	-	-	-
History of hepatitis B/C (positive / negative)	0.82	0.45-1.47	0.4969	-	-	-
History of malignancy (positive / negative)	1.25	0.84-1.86	0.2698	-	-	-
Body mass index (≥ 30 kg.m-2 / < 30 kg.m-2)	1.58	1.12-2.14	0.0084	-	-	-
Anti-class I PRA (positive / negative)	1.45	1.07-1.97	0.0182	-	-	-
Anti-class II PRA (positive / negative)	1.09	0.78-1.52	0.6299	-	-	-
Donor status (deceased/living)	2.50	1.41-4.43	0.0016	-	-	-
Donor EBV serology (positive / negative)	1.65	0.98-2.78	0.0606	-	-	-
Number of HLA-A-B-DR mismatches (> 4 / ≤ 4)	1.30	0.97-1.76	0.0824	-	-	-
Induction therapy (depleting / non depleting)	0.79	0.60-1.05	0.1091	-	-	-
Cold ischemia time (≥ 24 h / < 24 h)	1.29	1.01-1.66	0.0441	-	-	-
**Variables entering in both models**						
Transplantation period (< 2005 / ≥ 2005)	1.33	0.97-1.82	0.0693	0.97	0.55-1.74	0.9360
Recipient gender (male / female)	1.17	0.91-1.51	0.2186	0.61	0.38-1.05	0.0720
Recipient age (≥ 55 years / < 55 years)	1.39	1.05-1.83	0.0204	1.65	1.01-2.72	0.0480
Donor age (≥55 years / <55 years)	1.34	1.03-1.74	0.0313	0.59	0.33-0.99	0.0440
**Variables entering in the model for STR only**						
Donor gender (male / female)	-	-	-	1.53	1.03-2.48	0.0320
Waiting time before regraft ≥ 3 years	-	-	-	1.92	1.22-3.00	<0.0001

PRA, panel reactive antibody; EBV, Epstein-Barr virus; HLA, human leukocyte antigen.

### Relative hazard modelling in the STR group using the MRS

A first selection of variables was performed (p < 0.20) followed by a step-by-step descending procedure (Wald test with p < 0.05). In line with the requirements of additive-regression models, adjustments were forced for recipient gender and age and transplantation period. All the variables were categorized in order to avoid any log-linearity assumption and to obtain interpretable results.

The final relative model is presented in the last three columns of Table 2. Expected HR previously estimated in FTR are presented in the first columns to enable a direct comparison between FTR (Cox model) and STR (relative model). Donor gender and waiting time before retransplantation were not taken into account in the expected hazards for FTR. Donor gender was not a significant risk factor for FTR and waiting time is by definition a specific factor for STR. More precisely, we estimated a 1.5-fold increase in risk of graft failure for STR with grafts from males compared to STR with grafts from females (p = 0.0320). Moreover, STR who waited more than 3 years in dialysis before retransplantation had a 1.9-fold increased risk compared to STR with a shorter waiting time (p < 0.0001).

In contrast, the effect of recipient age and donor age seemed significantly different between FTR and STR (p < 0.05). More precisely, if we assumed a similar effect of recipient age between both groups, the expected HR associated with recipient age ≥ 55 years would be 1.39 in the STR group, regarding the HR observed in the FTR group. In fact, the relative model showed that this HR was 1.6-fold higher for STR compared to FTR (CI95% = [1.01-2.72], p = 0.0480). Similarly, the effect of donor age ≥ 55 years was nearly two fold lower for STR than for FTR (CI95% = [0.33-0.99], p = 0.0440), while it was identified as a significant risk factor for FTR (HR = 1.34, p = 0.0313). Of note, the relationship between the recipient gender and the risk of graft failure was not found to be significantly different between FTR and STR (p = 0.0720).

### Relative hazard modelling in the STR group using the SCM

As an alternative, we performed the SCM based on the same variables as those used in the previous MRS. Donor gender and waiting time before retransplantation were included in variables applied only in the relative part, i.e. *z*^*r*^. The other four variables (transplantation period, recipient gender and age, and donor age) were included in *z*^*c*^ and *z*^*s*^ to evaluate the difference in their effect between both groups. The results are presented in Table 3.

**Table 3:** Results of the stratified Cox model based on 2746 patients with 2206 FTR and 540 STR (26 STR presenting missing data for the waiting time before re-transplantation were excluded)

	**FTR strata**			**STR strata**		
	**HR**	**95% CI**	**p-value**	**HR**	**95% CI**	**p-value**
**Variables entering in z**^***e***^** only**						
Causal nephropathy (recurrent / non recurrent)	1.12	0.90-1.39	0.3031	-	-	-
History of diabetes (positive / negative)	1.28	0.95-1.72	0.1001	-	-	-
History of hypertension (positive / negative)	0.88	0.68-1.14	0.3270	-	-	-
History of cardiac disease (positive / negative)	1.35	1.10-1.66	0.0042	-	-	-
History of vascular disease (positive / negative)	1.11	0.85-1.47	0.4433	-	-	-
History of dyslipemia (positive / negative)	1.19	0.95-1.49	0.1263	-	-	-
History of hepatitis B/C (positive / negative)	0.97	0.65-1.46	0.9091	-	-	-
History of malignancy (positive / negative)	1.21	0.87-1.67	0.2646	-	-	-
Body mass index (≥ 30 kg.m-2 / < 30 kg.m-2)	1.54	1.13-2.08	0.0057	-	-	-
Anti-class I PRA (positive / negative)	1.39	1.07-1.82	0.0153	-	-	-
Anti-class II PRA (positive / negative)	0.98	0.73-1.31	0.8857	-	-	-
Donor status (deceased/living)	2.06	1.27-3.36	0.0036	-	-	-
Donor EBV serology (positive / negative)	1.65	1.07-2.54	0.0235	-	-	-
Number of HLA-A-B-DR mismatches (> 4 / ≤ 4)	1.33	1.01-1.75	0.0397	-	-	-
Induction therapy (depleting / non depleting)	0.88	0.70-1.11	0.2742	-	-	-
Cold ischemia time (≥ 24 h / < 24 h)	1.20	0.97-1.49	0.0894	-	-	-
**Variables entering in*z***^***c***^** and*z***^***s***^						
Transplantation period (< 2005 / ≥ 2005)	1.42	1.09-1.86	0.0099	0.94	0.54-1.64	0.8295
Recipient gender (male / female)	1.17	0.91-1.50	0.2200	0.63	0.40-1.02	0.0581
Recipient age (≥ 55 years / < 55 years)	1.36	1.03-1.78	0.0274	1.60	0.95-2.72	0.0785
Donor age (≥55 years / <55 years)	1.36	1.04-1.77	0.0238	0.60	0.35-1.05	0.0725
**Variables entering in*z***^***r***^** only**						
Donor gender (male / female)	-	-	-	1.51	0.97-2.36	0.0674
Waiting time before regraft ≥ 3 years	-	-	-	1.99	1.29-3.07	0.0019

PRA, panel reactive antibody; EBV, Epstein-Barr virus; HLA, human leukocyte antigen.

Estimations and corresponding 95% confidence intervals were very similar to those obtained in the MRS. Indeed, as in the previous model, we estimated a 2-fold increase in risk of graft failure for STR who waited more than 3 years in dialysis before retransplantation compared to STR who waited less than 3 years (p = 0.0019). The relationship between the donor gender and the risk of graft failure among STR was similar to that obtained in the MRS but was not found to be significant (HR = 1.51, p = 0.0674).

Transplantation period, recipient gender, recipient age and donor age were included in variables applied in both models. Results were also concordant with the MRS. For the four explicative variables, estimations and corresponding 95% confidence intervals were similar to those obtained in the MRS. However, conversely to the MRS, recipient age and donor age were not found to be significantly differently associated with the risk of graft failure between the two groups.

## Discussion

Although the comparison of survival between first and second kidney transplants has been frequently performed, no study has addressed the issue of comparing the risk factors associated with the time to graft failure between both groups. Understanding the factors influencing the long-term evolution of STR compared to FTR would benefit the medical management of graft attribution by identifying patients with the best chances.

The absence of literature focusing on this question may be partially explained by the methodological issues associated with such studies. Indeed, the Cox model is classically used to explore risk factors influencing graft survival and interactions can be included to evaluate risk factor differences between FTR and STR. However, this approach has several limitations. Firstly, it implies testing interactions between the graft rank and each explicative variable, increasing the number of parameters and making interpretations difficult. Secondly and certainly more importantly, only covariates common to both groups can be taken into account. This excludes explicative variables specific for one group. Concerning our application, this constitutes a limitation as several STR-specific explicative variables are known to be associated with second graft prognosis: the first graft transplantectomy [10], the first graft survival duration [6,11] or the time in dialysis before re-transplantation [5].

This paper describes two alternative models to overcome these difficulties. Firstly, the adaption of a multiplicative-regression model for relative survival allows a direct comparison of risk factors between two groups of patients without presupposing the role of each variable, i.e. common, different or specific relationships. The corresponding semi-parametric models are the Cox model for the expected hazard and the multiplicative-regression for the relative hazard. The main difficulty and limit of these models is the estimation of standard deviations which were obtained by Monte-Carlo simulations associated with bootstrap re-sampling. In this multiplicative modelling, the regression coefficients are straightforward to interpret in terms of their interactions. We propose an R package for a simple way of using the model.

Secondly, we demonstrated that a stratified Cox model specifying the graft rank as strata may be fitted to take into account STR-specific variables as a subvector of variables that enter in the model for STR only. In addition, some variables would enter either in the model for FTR only or in both models (with common or separate effects). The main limit to this approach is that the corresponding structure presupposes knowledge of variables potentially applicable to both models (in contrast with the relative model) unless testing a very large number of models. Indeed, whereas explicative variables entering in a single model (for FTR or STR) would easily be clinically assumed, those applicable to both models and with common or separate effects are not known in advance. Nevertheless, the SCM can be simply estimated by maximising a single partial likelihood function.

As expected, the results were concordant between both approaches. Regression coefficients were similar while standard deviations appeared a little smaller with the MRS approach. The results showed that male donor gender and a long waiting time before retransplantation were two specific-STR risk factors: donor gender was not significantly associated with the risk of graft failure in the FTR population and the waiting time before retransplantation was only related to STR by definition. The interpretations were similar to hazard ratios from a Cox model preformed on the STR group.

Conversely, two explicative variables appeared to be differently associated with the risk of graft failure between STR and FTR. More precisely, we showed for the first time that the adverse effect of recipient age was enhanced for STR as compared to FTR. The main clinical explanation is a cumulative effect of the risk factors for STR, in particular because of the cumulative exposure to immunosuppressive drugs during the first transplantation period. From a clinical point of view, this result may imply that clinicians should pay particular attention to recipient age in second kidney transplantations. Also, for the first time to our knowledge, this study identified an attenuation of the risk factor related to older transplants for STR as compared to FTR. Two explanations are: *(i)* an indication bias with only high-quality donors (without diabetes, hypertension or cardiovascular disease) proposed to STR; *(ii)* a higher non-HLA immunization in STR, explaining why graft failure is generally due to immunological phenomena rather than transplant quality.

Although we illustrated the advantages of both alternative approaches in renal transplantation, this methodology may be useful in number of other clinical and epidemiological applications. For practical use, we propose an R package to compute the MRS. The adaptation of the SCM can be computed by using many statistical software. Of course, the aim of such models is not to replace traditional survival models, but rather to provide a more suitable alternative when the main objective is to compare risk factors between two populations, in particular when population-specific covariates need to be included.

As always, there are several avenues worth exploring from this work. First, both models can be generalized for time-dependent explicative variables by adapting the likelihood functions as proposed by Therneau and Grambsch [27] (chapter 5, pages 111-115). Second, both models assumed the independence of FTR and STR. While this assumption was evaluated by using a frailty model among the 37 individuals common in both groups, a low statistical power may explain the non-rejection of this independence hypothesis. To ensure the validity of our results, we reperformed both models after exclusion of the corresponding 37 STR also included in FTR. The results presented in additional files showed the robustness of the results. Third, other strategies for variable selection can be adapted, such as partial likelihood generalization. Finally, further work is needed to develop a Goodness-of-fit statistic for the MRS approach, in particular concerning the proportional hazards assumption.

## Conclusions

MRS and SCM consitute two original approaches to compare risk factors between two populations. The advantage of MRS is to allow a direct modelling strategy but it is not straightforward to estimate the standard deviations. In contrast, SCM allows an overall estimation of parameters and standard deviations but its structure presupposes knowledge of the role of each explicative variable. This study also highlighted novel risk factor differences between first and second kidney transplant recipients. These results could help improve the management of patients waiting for a second graft. They may also encourage the widespread use of this original methodology in other medical fields.

## Competing interests

The authors declare that they have no competing interests.

## Authors’ contributions

KTL and YF designed the model. KTL carried out the statistical analysis and wrote the manuscript. YF performed statistical analysis, participated in writing the manuscript and extended the software to enable use of the method. MG and JD participated in research design, performed clinical analysis. MG and JD participated in writing of the manuscript. All authors read and approved the final manuscript.

## Pre-publication history

The pre-publication history for this paper can be accessed here:

http://www.biomedcentral.com/1471-2288/13/102/prepub

## Supplementary Material

Additional file 1 Multivariate Cox model for FTR (N = 2206) and results of the MRS in the STR group after exclusion of the 37 STR also included in FTR, based on 507 STR (22 recipients presenting missing data for the waiting time before re-transplantation were excluded). Click here for file

Additional file 2 Results of the stratified Cox model after exclusion of the 37 STR also included in FTR, based on 2713 patients with 2206 FTR and 507 STR (22 STR presenting missing data for the waiting time before re-transplantation were excluded). Click here for file
