# Supplementary material for: Comparison of the risk factors effects between two populations: two alternative approaches illustrated by the analysis of first and second kidney transplant recipients
Source: BMC Med Res Methodol. 2013 Aug 6;13:102. doi: 10.1186/1471-2288-13-102 (PMC3750352; doi:10.1186/1471-2288-13-102)
Supplement: Additional file 1 — Multivariate Cox model for FTR (N = 2206) and results of the MRS in the STR group after exclusion of the 37 STR also included in FTR, based on 507 STR (22 recipients presenting missing data for the waiting time before re-transplantation were excluded). [file 1471-2288-13-102-S1.pdf]

Additional file 1 — Multivariate Cox model for FTR (N = 2206) and results of the MRS in the STR group after exclusion of the 37 STR also included in FTR, based on 507 STR (22 recipients presenting missing data for the waiting time before re-transplantation were excluded)

	Cox model in the FTR group			MRS in the STR group		
	HR	95% CI	p-value	HR	95% CI	p-value
**Variables entering in the model for FTR only**						
Causal nephropathy (recurrent / non recurrent)	1.24	0.96-1.59	0.0987	-	-	-
History of diabetes (positive / negative)	1.34	0.96-1.85	0.0819	-	-	-
History of hypertension (positive / negative)	0.77	0.57-1.05	0.0986	-	-	-
History of cardiac disease (positive / negative)	1.41	1.11-1.79	0.0051	-	-	-
History of vascular disease (positive / negative)	1.10	0.81-1.51	0.5351	-	-	-
History of dyslipemia (positive / negative)	1.12	0.87-1.45	0.3828	-	-	-
History of hepatitis B/C (positive / negative)	0.82	0.45-1.47	0.4969	-	-	-
History of malignancy (positive / negative)	1.25	0.84-1.86	0.2698	-	-	-
Body mass index ($\geq$ 30 kg.m-2 / $<$ 30 kg.m-2)	1.58	1.12-2.14	0.0084	-	-	-
Anti-class I PRA (positive / negative)	1.45	1.07-1.97	0.0182	-	-	-
Anti-class II PRA (positive / negative)	1.09	0.78-1.52	0.6299	-	-	-
Donor status (deceased/living)	2.50	1.41-4.43	0.0016	-	-	-
Donor EBV serology (positive / negative)	1.65	0.98-2.78	0.0606	-	-	-
Number of HLA-A-B-DR mismatches ($>$ 4 / $\leq$ 4)	1.30	0.97-1.76	0.0824	-	-	-
Induction therapy (depleting / non depleting)	0.79	0.60-1.05	0.1091	-	-	-
Cold ischemia time ($\geq$ 24 h / $<$ 24 h)	1.29	1.01-1.66	0.0441	-	-	-
**Variables entering in both models**						
Transplantation period ($<$ 2005 / $\geq$ 2005)	1.33	0.97-1.82	0.0693	1.02	0.63-1.67	0.9254
Recipient gender (male / female)	1.17	0.91-1.51	0.2186	0.63	0.41-0.95	0.0290
Recipient age ($\geq$ 55 years / $<$ 55 years)	1.39	1.05-1.83	0.0204	1.47	0.92-2.33	0.1032
Donor age ($\geq$55 years / $<$55 years)	1.34	1.03-1.74	0.0313	0.60	0.37-0.98	0.0416
**Variables entering in the model for STR only**						
Donor gender (male / female)	-	-	-	1.47	0.94-2.30	0.0948
Waiting time before regraft $\geq$ 3 years	-	-	-	1.84	1.19-2.84	0.0061

PRA, panel reactive antibody; EBV, Epstein-Barr virus; HLA, human leukocyte antigen
