# Supplementary material for: Comparison of the risk factors effects between two populations: two alternative approaches illustrated by the analysis of first and second kidney transplant recipients
Source: BMC Med Res Methodol. 2013 Aug 6;13:102. doi: 10.1186/1471-2288-13-102 (PMC3750352; doi:10.1186/1471-2288-13-102)
Supplement: Additional file 2 — Results of the stratified Cox model after exclusion of the 37 STR also included in FTR, based on 2713 patients with 2206 FTR and 507 STR (22 STR presenting missing data for the waiting time before re-transplantation were excluded). [file 1471-2288-13-102-S2.pdf]

Additional file 2 — Results of the stratified Cox model after exclusion of the 37 STR also included in FTR, based on 2713 patients with 2206 FTR and 507 STR (22 STR presenting missing data for the waiting time before re-transplantation were excluded)

	FTR strata			STR strata		
	HR	95% CI	p-value	HR	95% CI	p-value
**Variables entering in z^e only**						
Causal nephropathy (recurrent / non recurrent)	1.13	0.91-1.40	0.2792	-	-	-
History of diabetes (positive / negative)	1.26	0.93-1.69	0.1330	-	-	-
History of hypertension (positive / negative)	0.89	0.68-1.15	0.3593	-	-	-
History of cardiac disease (positive / negative)	1.36	1.10-1.67	0.0041	-	-	-
History of vascular disease (positive / negative)	1.13	0.85-1.49	0.3949	-	-	-
History of dyslipemia (positive / negative)	1.20	0.96-1.51	0.1174	-	-	-
History of hepatitis B/C (positive / negative)	0.98	0.65-1.47	0.9263	-	-	-
History of malignancy (positive / negative)	1.20	0.86-1.68	0.2744	-	-	-
Body mass index ($\geq$ 30 kg.m-2 / $<$ 30 kg.m-2)	1.53	1.13-2.08	0.0066	-	-	-
Anti-class I PRA (positive / negative)	1.43	1.09-1.87	0.0090	-	-	-
Anti-class II PRA (positive / negative)	0.98	0.73-1.31	0.8761	-	-	-
Donor status (deceased/living)	2.04	1.25-3.32	0.0042	-	-	-
Donor EBV serology (positive / negative)	1.66	1.08-2.55	0.0219	-	-	-
Number of HLA-A-B-DR mismatches ($>$ 4 / $\leq$ 4)	1.34	1.02-1.77	0.0345	-	-	-
Induction therapy (depleting / non depleting)	0.89	0.71-1.12	0.3138	-	-	-
Cold ischemia time ($\geq$ 24 h / $<$ 24 h)	1.22	0.99-1.51	0.0656	-	-	-
**Variables entering in z^c and z^s**						
Transplantation period ($<$ 2005 / $\geq$ 2005)	1.41	1.08-1.85	0.0113	0.99	0.56-1.74	0.9848
Recipient gender (male / female)	1.17	0.91-1.50	0.2112	0.64	0.39-1.03	0.0664
Recipient age ($\geq$ 55 years / $<$ 55 years)	1.36	1.04-1.78	0.0271	1.48	0.86-2.53	0.1564
Donor age ($\geq$55 years / $<$55 years)	1.36	1.04-1.77	0.0238	0.62	0.35-1.09	0.0725
**Variables entering in z^r only**						
Donor gender (male / female)	-	-	-	1.47	0.94-2.31	0.0941
Waiting time before regraft $\geq$ 3 years	-	-	-	1.90	1.22-2.97	0.0047

PRA, panel reactive antibody; EBV, Epstein-Barr virus; HLA, human leukocyte antigen
